# Supplementary material for: The Skin We Live in: Pigmentation Traits and Tanning Behaviour in British Young Adults, an Observational and Genetically-Informed Study
Source: Genes (Basel). 2022 May 17;13(5):896. doi: 10.3390/genes13050896 (PMC9140533; doi:10.3390/genes13050896)

Supplementary Figure S1. Connections between text answer categories of reasons for liking to tan reported by ALSPAC young people (YP) in the Tanning and Sun Exposure section of the Life@25+ questionnaire. Statistically significant coincidences are represented by dotted lines ($p < 0.05$) or a continuous line ($p < 0.001$).

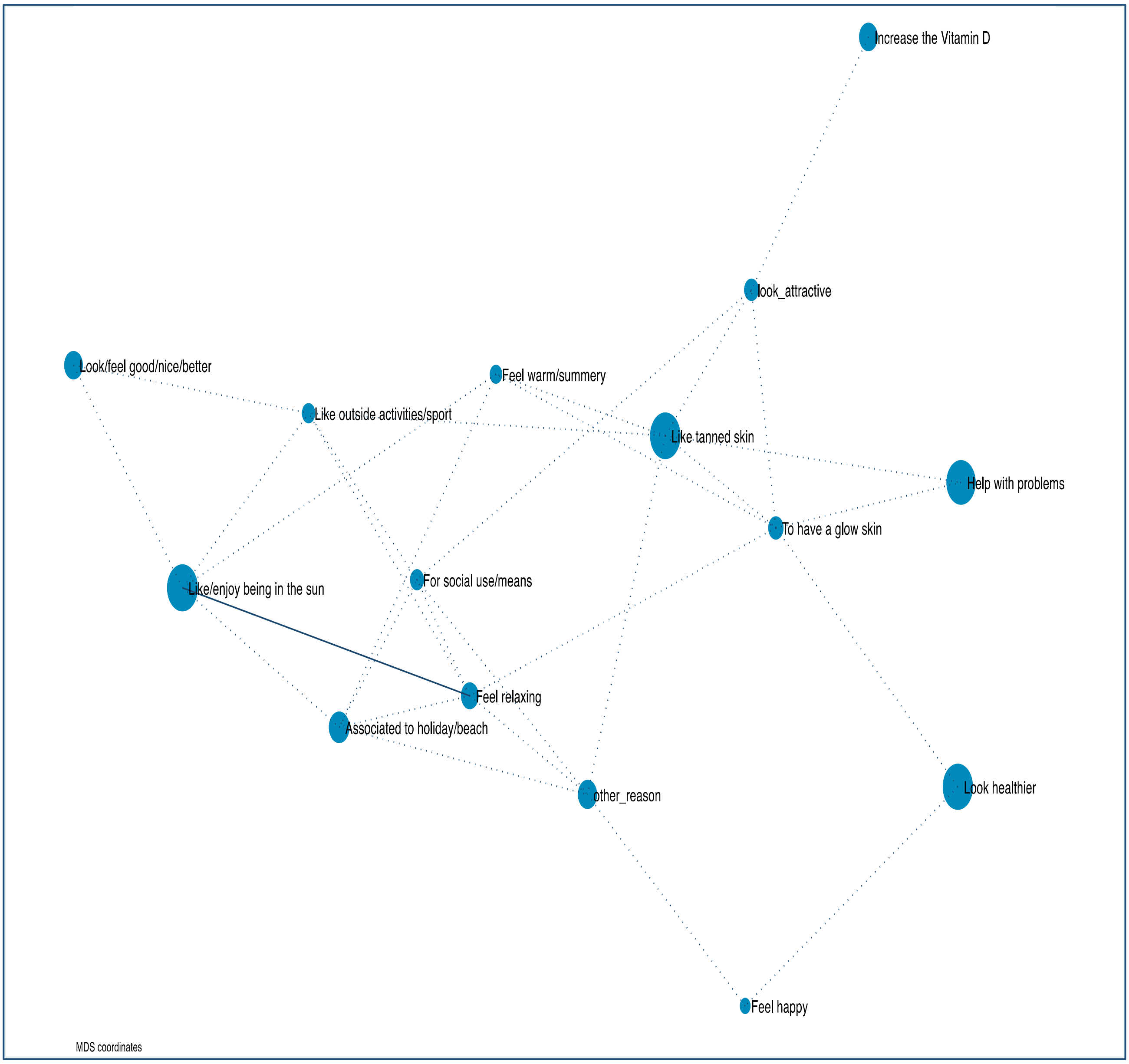

Supplementary Figure S2. Skin and hair colour distribution among ALSPAC young people (YP) who completed the Tanning and Sun Exposure section of the Life@25+ questionnaire.

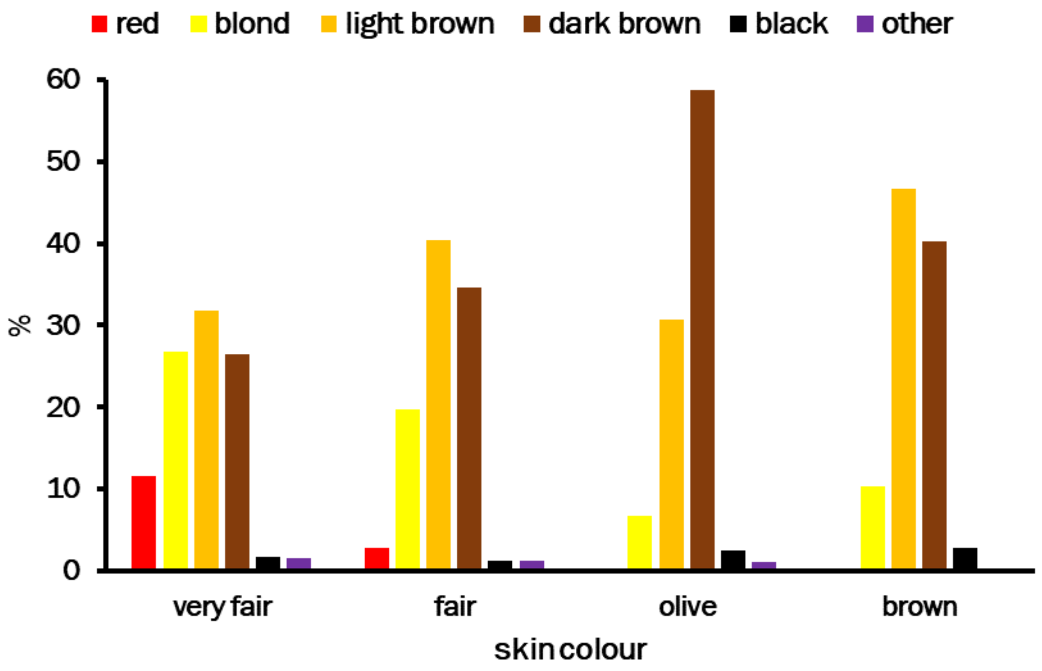

Supplementary Figure S3. Skin and eye colour distribution among ALSPAC young people (YP) who completed the Tanning and Sun Exposure section of the Life@25+ questionnaire.

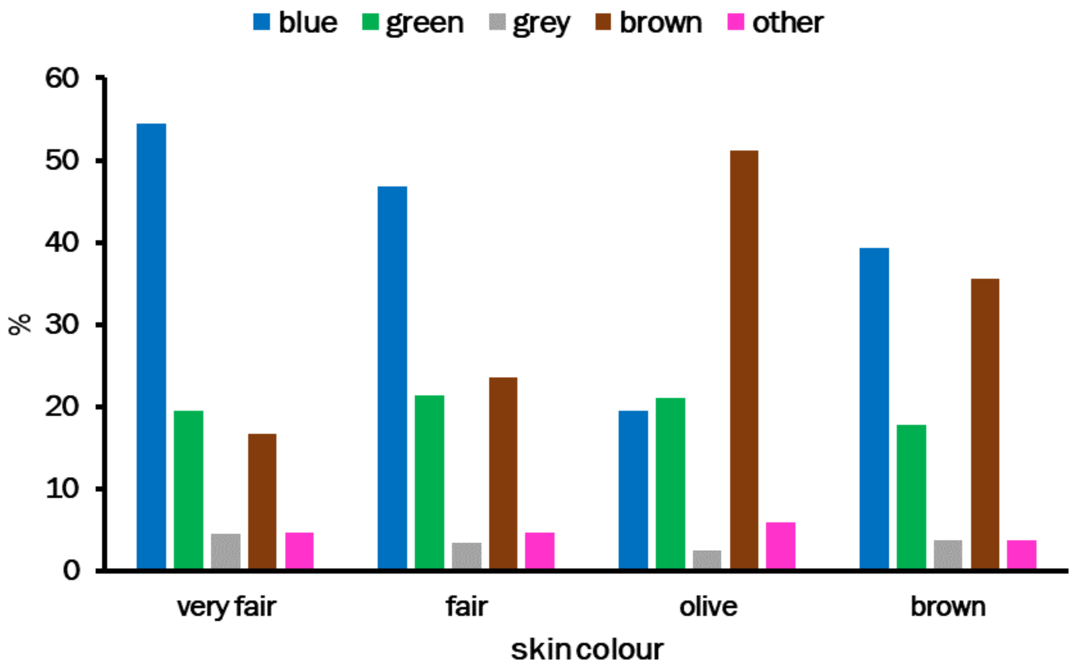

Supplementary Figure S4. Hair and eye colour distribution among ALSPAC young people (YP) who completed the Tanning and Sun Exposure section of the Life@25+ questionnaire.

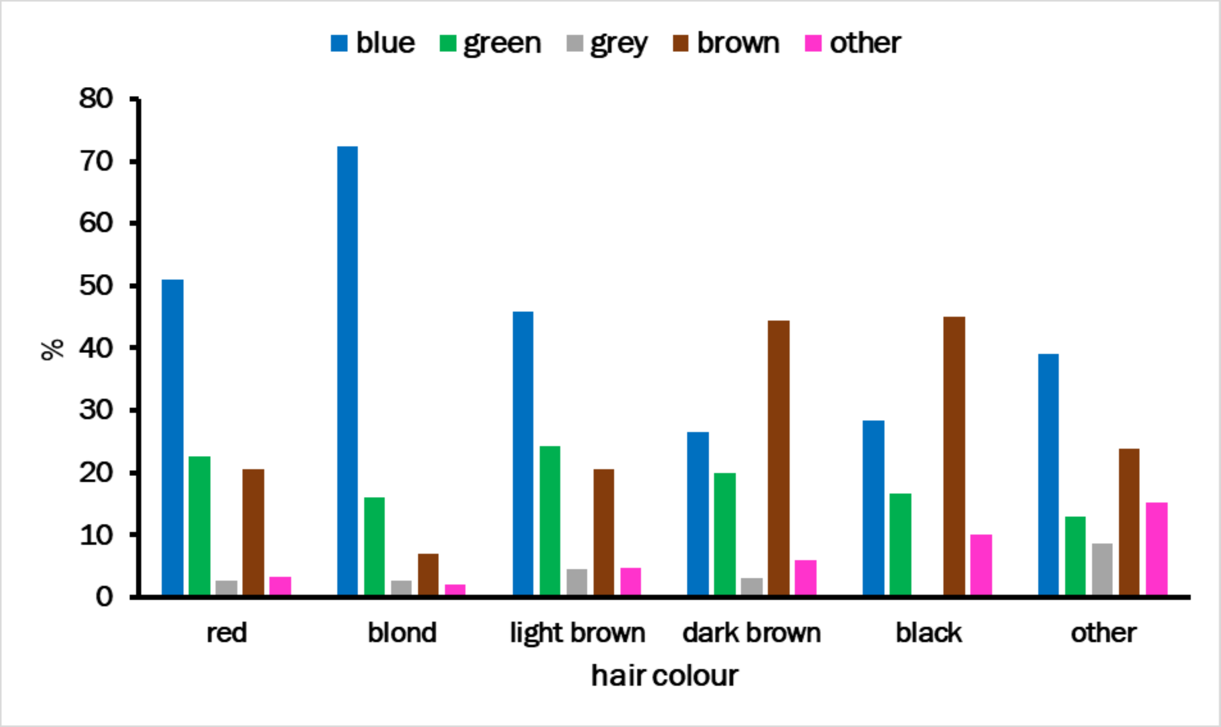

Supplementary Figure S5. Hierarchical clustering visualization of sun protection strategies reported as text answers by ALSPAC young people (YP) in the Tanning and Sun Exposure section of the Life@25+ questionnaire.

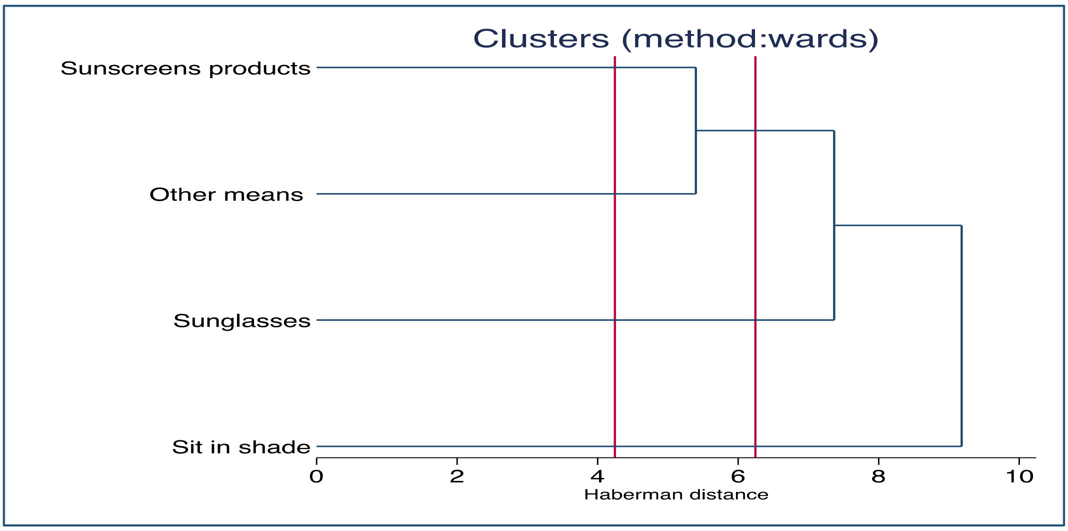

Supplement: Supplementary file 1 [file genes-13-00896-s001.zip › Bonilla&Mejia-supplementary_figures-rev.pdf]
