# Supplementary material for: The Skin We Live in: Pigmentation Traits and Tanning Behaviour in British Young Adults, an Observational and Genetically-Informed Study
Source: Genes (Basel). 2022 May 17;13(5):896. doi: 10.3390/genes13050896 (PMC9140533; doi:10.3390/genes13050896)
Supplement: Supplementary file 1 [file genes-13-00896-s001.zip › Bonilla&Mejia-supplementary_tables-rev.pdf]

Supplementary Table S2. Best area under the curve (AUC) at a population level and mean (standard deviation) loss of AUC for each predicted trait in ALSPAC young people (YP) (N = 8564).

trait	full AUC	loss of AUC
blue eyes	0.939	0.00042 (0.00098)
intermediate eyes	0.736	0.00172 (0.00398)
brown eyes	0.946	0.00071 (0.00167)
blond hair	0.813	0.00403 (0.00445)
brown hair	0.741	0.00252 (0.00390)
red hair	0.929	0.00858 (0.01692)
black hair	0.859	0.00196 (0.00341)
hair shade	0.905	0.00160 (0.00297)
very pale skin	0.830	0.00101 (0.00314)
pale skin	0.763	0.00225 (0.00335)
intermediate skin	0.783	0.00307 (0.00434)
dark skin	0.981	0.00087 (0.00207)
dark to black skin	0.993	0.00051 (0.00081)

Supplementary Table S3. Text answers of ALSPAC young people (YP) who responded "other" to the question "Why do you like to tan?". Percentages over the total number of answers are shown.

reasons	n = 331 answers	%
like or enjoy being under the sun	49	14.8
like tanned skin	48	14.5
look or be healthier	47	14.2
helps with skin problems such as acne, eczema, spots	44	13.3
associated to holidays or beach	21	6.3
increase vitamin D	17	5.2
look or feel good/nice	17	5.2
is relaxing/enjoyable	15	4.5
social reasons & professional reasons	12	3.6
have glowing skin	11	3.3
look more attractive	10	3.0
due to outside activities/sports	8	2.4
feel warm or summery	7	2.1
feel happy	5	1.5
other reasons (YP is naturally tanned, just happens, likes freckles, no special reason, does it sometimes, why not do that, photos/shoots, does not do it frequently)	20	6.0

Supplementary Table S4. Text answers of ALSPAC young people (YP) who reported having “other” hair colour. Percentages over the total number of answers are shown.

hair colour category	n = 79 answers	%
red	26	32.9
blond	15	19.0
ginger	14	17.7
brown	12	15.2
auburn	7	8.9
other	5	6.3

Supplementary Table S5. Text answers of ALSPAC young people (YP) who reported having “other” eye colour. Percentages over the total number of answers are shown.

eye colour category	n = 257 answers	%
hazel	110	42.8
green	56	21.8
blue	35	13.6
black/brown	27	10.5
grey	14	5.4
other	10	3.9
changing	5	1.9

Supplementary Table S7. Text answers of ALSPAC young people (YP) who reported taking "other" measures for sun protection. Percentages over the total number of answers are shown.

sun protective measures	n = 48 answers	%
sit in the shade	13	27.1
use protection sometimes & frequent breaks from the sun & physical barriers such as clothing	9	18.8
sunscreen products	8	16.7
sunglasses	6	12.5
moisturizing products & tanning oils	6	12.5
other measures (drink water, wear make-up, depends how hot it is, all of the above, lip protection)	6	12.5

Supplementary Table S8. Factors associated with reasons for liking to tan. Only those with the strongest evidence for association are shown.

reason for tanning	it gives YP more confidence[a]	it makes YP feel happier[a]	makes YP look better in photos[a]	makes YP look thinner[a]	conceals body imperfections[a]	YP looks more attractive to others[a]	pale skin is unattractive[a]
**sex** (0=female/1=male) (N=1930)	0.34 (0.27,0.42), < 10^{-26}	0.44 (0.35,0.54), 1.35×10^{-13}	0.67 (0.54,0.82), 1.57×10^{-4}	0.05 (0.03,0.09), < 10^{-26}	0.14 (0.09,0.20), < 10^{-26}	1.17 (0.95,1.44), 0.136	0.81 (0.62,1.05), 0.106
**mother's social class** (0=manual/1=non-manual) (N=1598)	1.33 (0.98,1.80), 0.068	1.19 (0.86,1.64), 0.290	0.94 (0.70,1.26), 0.695	1.04 (0.74,1.45), 0.837	1.23 (0.88,1.72), 0.223	1.27 (0.95,1.70), 0.109	0.61 (0.44,0.84), 0.003
**father's social class** (0=manual/1=non-manual) (N=1704)	0.96 (0.78,1.20), 0.743	1.02 (0.81,1.27), 0.875	1.10 (0.90,1.34), 0.357	1.00 (0.79,1.27), 0.996	1.00 (0.79,1.25), 0.973	1.26 (1.03,1.53), 0.025	0.78 (0.61,0.99), 0.041
**maternal education** (N=1814) (model p-value)[1]	0.834	0.250	0.989	0.578	0.099	0.007	0.039
O level	1.09 (0.82,1.45), 0.548	1.19 (0.88,1.60), 0.260	1.02 (0.78,1.33), 0.896	0.99 (0.73,1.36), 0.971	1.33 (0.97,1.81), 0.078	1.33 (1.02,1.74), 0.037	0.77 (0.57,1.05), 0.105
> O level	1.05 (0.80,1.38), 0.717	0.98 (0.74,1.29), 0.866	1.00 (0.78,1.29), 0.972	0.88 (0.65,1.20), 0.423	1.38 (1.02,1.87), 0.034	1.50 (1.16,1.93), 0.002	0.68 (0.51,0.91), 0.011
**skin colour** (N=1929) (model p-value)[2]	0.023	0.038	0.013	0.386	0.039	0.004	4.99×10^{-5}
fair	0.64 (0.45,0.89), 0.008	1.31 (0.96,1.79), 0.093	0.74 (0.55,1.00), 0.051	0.85 (0.61,1.17), 0.318	0.73 (0.53,0.99), 0.044	0.66 (0.49,0.88), 0.005	0.55 (0.41,0.76), < 2.31×10^{-4}

olive	0.81 (0.56,1.16), 0.249	1.58 (1.11,2.24), 0.011	0.92 (0.66,1.28), 0.633	0.91 (0.63,1.31), 0.622	0.83 (0.59,1.18), 0.297	0.78 (0.57,1.08), 0.130	0.44 (0.31,0.64), < 1.15×10^{-5}
brown	0.64 (0.36,1.14), 0.130	1.98 (1.07,3.68), 0.030	0.49 (0.28,0.84), 0.009	0.54 (0.25,1.14), 0.107	0.39 (0.18,0.82), 0.013	0.43 (0.25,0.74), 0.003	0.33 (0.16,0.68), 0.003
**tanning ability** (N=1923) (model p-value)[3]	0.023	0.004	0.035	0.760	0.080	0.049	4.78×10^{-7}
burns easily rarely tans	0.61 (0.23,1.67), 0.339	0.10 (0.01,0.77), 0.027	0.44 (0.17,1.12), 0.085	0.80 (0.35,1.81), 0.593	0.44 (0.2,0.98), 0.044	0.80 (0.36,1.76), 0.574	0.50 (0.23,1.10), 0.085
doesn't change	0.68 (0.23,2.05), 0.496	0.11 (0.01,0.83), 0.033	0.40 (0.14,1.09), 0.074	1.07 (0.42,2.76), 0.887	0.37 (0.15,0.95), 0.038	0.69 (0.28,1.68), 0.409	0.36 (0.14,0.92), 0.033
tans easily rarely burns	0.54 (0.20,1.45), 0.220	0.13 (0.02,0.98), 0.048	0.41 (0.16,1.03), 0.057	0.77 (0.34,1.71), 0.518	0.39 (0.18,0.84), 0.017	0.61 (0.28,1.33), 0.212	0.26 (0.12,0.56), 5.76×10^{-4}
always tans never burns	0.74 (0.25,2.21), 0.593	0.13 (0.02,1.02), 0.052	0.38 (0.14,1.04), 0.059	0.66 (0.25,1.72), 0.395	0.47 (0.19,1.16), 0.100	0.42 (0.17,1.03), 0.059	0.30 (0.12,0.75), 0.010
can't say, skin always protected	0.22 (0.07,0.71), 0.011	0.05 (0.01,0.41), 0.005	0.17 (0.06,0.51), 0.002	0.62 (0.21,1.87), 0.398	0.20 (0.06,0.61), 0.005	0.54 (0.20,1.43), 0.214	0.23 (0.07,0.68), 0.009
**sunburning past 2 years** (N=1868) (model p-value)[4]	0.041	0.190	1.79×10^{-4}	0.053	0.112	1.52×10^{-6}	0.012
once	1.35 (1.02,1.80), 0.037	0.82 (0.61,1.11), 0.196	1.60 (1.22,2.09), 6.24×10^{-4}	1.28 (0.92,1.78), 0.138	1.31 (0.96,1.80), 0.086	1.51 (1.15,1.98), 0.003	1.28 (0.91,1.81), 0.160
twice	1.18 (0.90,1.56), 0.235	0.77 (0.57,1.03), 0.073	1.29 (0.99,1.67), 0.059	1.24 (0.89,1.71), 0.200	1.20 (0.88,1.64), 0.247	1.65 (1.26,2.15), 2.37×10^{-4}	1.47 (1.06,2.06), 0.023

3times	1.56 (1.08,2.26), 0.018	1.13 (0.76,1.68), 0.544	2.03 (1.43,2.88), $6.62x10^{-5}$	1.44 (0.96,2.17), 0.077	1.69 (1.15,2.48), 0.007	2.06 (1.46,2.90), $3.37x10^{-5}$	1.72 (1.15,2.60), 0.009
4times	1.49 (0.96,2.29), 0.073	1.08 (0.68,1.70), 0.756	1.65 (1.10,2.47), 0.015	1.24 (0.76,2.03), 0.395	1.36 (0.85,2.16), 0.196	2.68 (1.78,4.03), $2.21x10^{-6}$	1.27 (0.77,2.10), 0.349
5times or more	1.98 (1.16,3.36), 0.012	0.97 (0.57,1.66), 0.924	2.08 (1.28,3.37), 0.003	2.39 (1.40,4.11), 0.002	1.63 (0.96,2.77), 0.069	1.93 (1.21,3.08), 0.006	2.43 (1.44,4.10), $9.19x10^{-4}$
**YP does not use sun protection** (N=1928)	0.92 (0.64,1.34), 0.667	0.67 (0.47,0.97), 0.035	0.84 (0.59,1.20), 0.341	1.06 (0.68,1.64), 0.799	1.24 (0.83,1.86), 0.297	1.29 (0.91,1.82), 0.160	1.82 (1.24,2.65), 0.002
**wears sunblock** (N=1928)	1.47 (1.01,2.14), 0.046	1.38 (0.94,2.02), 0.103	1.64 (1.13,2.36), 0.008	1.07 (0.67,1.71), 0.764	1.23 (0.79,1.94), 0.362	0.93 (0.65,1.34), 0.690	0.73 (0.48,1.10), 0.132
**wears a hat** (N=1928)	0.93 (0.75,1.17), 0.550	1.19 (0.93,1.50), 0.161	0.97 (0.78,1.20), 0.768	0.95 (0.73,1.23), 0.689	0.93 (0.73,1.19), 0.572	0.74 (0.60,0.91), 0.005	0.85 (0.66,1.10), 0.225
**tanning helps prevent sunburn** (N=1926) (model p-value)[5]	$1.76x10^{-4}$	0.001	$4.31x10^{-6}$	$8.42x10^{-6}$	0.006	0.044	$3.91x10^{-5}$
yes	2.06 (1.47,2.90), $3.21x10^{-5}$	1.95 (1.36,2.80), $2.56x10^{-4}$	2.18 (1.60,2.98), $7.29x10^{-7}$	2.07 (1.51,2.83), $6.31x10^{-6}$	1.65 (1.21,2.24), 0.001	1.39 (1.05,1.84), 0.023	2.02 (1.49,2.74), $6.95x10^{-6}$
don't know	1.08 (0.84,1.39), 0.529	1.13 (0.87,1.46), 0.373	1.05 (0.83,1.33), 0.694	0.87 (0.65,1.18), 0.381	1.13 (0.85,1.49), 0.369	0.93 (0.74,1.17), 0.532	1.09 (0.82,1.46), 0.534
**indoor tanning can cause skin cancer** (N=1924) (model p-value)[5]	0.005	0.013	0.348	$2.36x10^{-4}$	0.005	0.001	0.107
yes	1.16 (0.64,2.10), 0.631	1.45 (0.81,2.62), 0.213	0.98 (0.55,1.72), 0.931	0.64 (0.35,1.18), 0.155	0.90 (0.49,1.65), 0.732	1.03 (0.59,1.79), 0.921	0.61 (0.34,1.10), 0.101

don’t know	0.72 (0.37,1.39), 0.326	1.26 (0.65,2.45), 0.489	0.58 (0.31,1.09), 0.091	0.25 (0.12,0.54), 3.88×10^{-4}	0.45 (0.21,0.92), 0.030	0.54 (0.29,1.02), 0.057	0.47 (0.24,0.95), 0.035
**YP trying to change skin colour** (N=1537)	2.36 (1.54,3.62), 8.55×10^{-5}	1.62 (1.07,2.47), 0.023	1.92 (1.34,2.76), 3.75×10^{-4}	2.06 (1.45,2.94), 5.95×10^{-5}	1.69 (1.19,2.39), 0.003	1.73 (1.24,2.42), 0.001	1.79 (1.25,2.56), 0.001
**YP trying to change hair colour** (N=1537)	1.48 (0.94,2.35), 0.094	1.53 (0.93,2.51), 0.091	2.14 (1.38,3.31), 7.05×10^{-4}	1.99 (1.32,3.00), 0.001	2.09 (1.39,3.13), 3.52×10^{-4}	1.25 (0.85,1.85), 0.264	1.41 (0.91,2.19), 0.123
**YP’s teachers treat everybody the same** (N=1478)	1.51 (0.82,2.78), 0.189	1.41 (0.74,2.68), 0.300	0.90 (0.52,1.54), 0.695	1.62 (0.85,3.10), 0.146	1.44 (0.77,2.68), 0.253	0.63 (0.36,1.10), 0.104	2.04 (1.15,3.63), 0.015

[a]OR (95% CI), p-value, from logistic regression model adjusted for age and sex. YP = young person.

[1]reference: < O level

[2]reference: very fair

[3]reference: always burns, never tans

[4]reference: never

[5]reference: no

Supplementary Table S9. Association of socio-demographic, pigmentation-related and sun exposure factors with outdoor tanning in ALSPAC young people (YP) who like to tan.

exposure	OR[a]	95% CI	p-value	N
**sex** (0=female/1=male)	6.11	(3.29,11.32)	9.35×10^{-9}	1929
**age** (years)	1.25	(0.96,1.62)	0.103	1929
**mother's social class** (0=manual/1=non-manual)	1.66	(1.09,2.52)	0.018	1597
**father's social class** (0=manual/1=non-manual)	1.60	(1.15,2.21)	0.005	1703
**mother's education**			3.83×10^{-5}	1813
< O level	reference			
O level	1.68	(1.14,2.47)	0.009	
> O level	2.44	(1.66,3.61)	6.55×10^{-6}	
**skin colour**			2.71×10^{-12}	1928
very fair	reference			
fair	3.09	(2.14,4.44)	1.35×10^{-9}	
olive	5.30	(3.25,8.66)	2.66×10^{-11}	
brown	9.42	(2.22,40.03)	0.002	
**eye colour**			0.942	1925
blue	reference			
green	1.07	(0.72,1.58)	0.748	
grey	1.56	(0.55,4.47)	0.405	
brown	1.06	(0.74,1.52)	0.766	
other	0.99	(0.49,2.00)	0.980	
**natural hair colour at 18 years old**			8.39×10^{-6}	1908
red	reference			
blond	3.72	(1.85,7.44)	2.14×10^{-4}	
light brown	5.10	(2.60,9.98)	2.02×10^{-6}	
dark brown	5.94	(3.00,11.80)	3.43×10^{-7}	

black	1.00	n/a	n/a	
other	3.32	(0.96,11.50)	0.058	
**YP has freckles**			0.004	1925
no	reference			
yes, a few	0.62	(0.44,0.87)	0.006	
yes, many	0.51	(0.32,0.80)	0.003	
**tanning ability**			1.69×10^{-17}	1922
always burns never tans	reference			
burns easily rarely tans	8.24	(3.50,19.41)	1.37×10^{-6}	
doesn't change	4.69	(1.75,12.55)	0.002	
tans easily rarely burns	23.79	(10.13,55.86)	3.41×10^{-13}	
always tans never burns	14.21	(4.72,42.79)	2.40×10^{-6}	
can’t say, skin always protected	14.78	(3.93,55.58)	6.72×10^{-5}	
**sun burning in the past 2 years**			0.022	1867
never	reference			
once	1.32	(0.88,1.98)	0.178	
twice	1.93	(1.25,2.97)	0.003	
3 times	1.89	(1.06,3.38)	0.031	
4 times	1.57	(0.80,3.07)	0.186	
5 times or more	0.84	(0.43,1.64)	0.611	
**sun protection** (0=no/1=yes)				
never uses protection	0.70	(0.41,1.20)	0.193	1927
wears hat	1.21	(0.83,1.75)	0.317	1927
wears clothing	0.89	(0.63,1.25)	0.496	1927
wears sunblock	1.35	(0.76,2.40)	0.298	1927
avoids the sun	0.83	(0.50,1.38)	0.463	1927
**sunblock factor**			0.162	1797
lower than 15	reference			

15-24	0.94	(0.49,1.80)	0.856	
25-49	0.80	(0.42,1.52)	0.492	
50 or higher	0.52	(0.24,1.11)	0.092	
**skin cancer** (0=no/1=yes)				
YP has family member diagnosed with skin cancer	0.82	(0.54,1.24)	0.339	1924
**beliefs about tanning**				
YP believes indoor tanning helps prevent sunburn			0.016	1925
no	reference			
yes	0.55	(0.37,0.83)	0.004	
don't know	0.86	(0.58,1.28)	0.452	
YP thinks indoor tanning using sun bed/sun lamp can cause skin cancer			0.580	1923
no	reference			
yes	1.51	(0.69,3.30)	0.300	
don't know	1.44	(0.57,3.60)	0.437	
**body image**				
YP is trying to change skin colour (0=no/1=yes)	0.84	(0.50,1.39)	0.490	1536
YP is trying to change hair colour (0=no/1=yes)	0.60	(0.35,1.03)	0.063	1536
YP has been hurt because of their skin colour (0=no/1=yes)	0.66	(0.08,5.42)	0.695	1603
YP has been called names because of their skin colour (0=no/1=yes)	0.50	(0.18,1.36)	0.175	1607
YP has seen someone bullied because of their skin colour (0=no/1=yes)	1.07	(0.73,1.57)	0.741	1598
YP's teachers treat everybody the same, regardless of skin colour (0=agree/1=disagree)	0.70	(0.29,1.71)	0.438	1477

[a]Adjusted for age and sex.

Supplementary Table S10. Association of socio-demographic, pigmentation-related and sun exposure factors with indoor tanning in the past 12 months amongst ALSPAC young people (YP) who manifested ever engaging in indoor tanning using a sun bed/sun lamp/tanning booth.

**exposure**	**OR[a]**	**95% CI**	**p-value**	**N**
**sex** (0=female/1=male)	0.79	(0.54,1.15)	0.216	946
**age** (years)	0.83	(0.66,1.03)	0.095	946
**mother's social class** (0=manual/1=non-manual)	1.01	(0.69,1.47)	0.970	775
**father's social class** (0=manual/1=non-manual)	0.61	(0.46,0.80)	4.46×10^{-4}	837
**mother's education**			0.014	884
< O level	reference			
O level	1.18	(0.83,1.67)	0.356	
> O level	0.75	(0.53,1.06)	0.108	
**skin colour**			0.003	943
very fair	reference			
fair	1.60	(1.08,2.38)	0.019	
olive	2.24	(1.44,3.50)	3.57×10^{-4}	
brown	2.60	(1.03,6.55)	0.043	
**eye colour**			0.131	945
blue	reference			
green	1.47	(1.05,2.06)	0.025	
grey	2.06	(0.88,4.82)	0.096	
brown	1.21	(0.89,1.65)	0.227	
other	1.08	(0.56,2.09)	0.823	
**natural hair colour at 18 years old**			0.725	946
red	reference			
blonde	1.97	(0.65,6.00)	0.231	
light brown	1.95	(0.65,5.81)	0.233	

dark brown	2.00	(0.67,6.01)	0.215	
black	0.60	(0.05,7.03)	0.681	
other	2.62	(0.48,14.35)	0.267	
**YP has freckles**			0.044	944
no	reference			
yes, a few	1.15	(0.87,1.51)	0.333	
yes, many	0.69	(0.46,1.04)	0.076	
**tanning ability**			1.65×10^{-4}	939
always burns never tans	reference			
burns easily rarely tans	2.25	(0.97,5.22)	0.060	
doesn't change	1.83	(0.64,5.26)	0.260	
tans easily rarely burns	3.51	(1.55,7.99)	0.003	
always tans never burns	7.11	(2.55,19.81)	1.73×10^{-4}	
can't say, skin always protected	2.62	(0.69,9.98)	0.159	
**sun burning in the past 2 years**			0.132	906
never	reference			
once	0.71	(0.49,1.02)	0.066	
twice	0.63	(0.43,0.92)	0.017	
3 times	0.72	(0.44,1.17)	0.182	
4 times	1.07	(0.59,1.93)	0.832	
5 times or more	0.99	(0.53,1.83)	0.964	
**sun protection** (0=no/1=yes)				
never uses protection	1.28	(0.77,2.11)	0.337	944
wears hat	0.83	(0.62,1.13)	0.244	944
wears clothing	0.68	(0.50,0.94)	0.019	944
wears sunblock	0.79	(0.47,1.34)	0.390	944
avoids the sun	0.55	(0.33,0.91)	0.020	944
**sunblock factor**			1.18×10^{-7}	883

lower than 15	reference			
15-24	0.75	(0.47,1.20)	0.231	
25-49	0.42	(0.26,0.67)	2.72×10^{-4}	
50 or higher	0.20	(0.10,0.41)	8.39×10^{-6}	
**skin cancer** (0=no/1=yes)				
YP has family member diagnosed with skin cancer	1.13	(0.76,1.67)	0.548	944
**beliefs about tanning** (0=no/1=yes)				
YP believes indoor tanning helps prevent sunburn			9.04×10^{-14}	946
no	reference			
yes	4.10	(2.87,5.86)	1.04×10^{-14}	
don't know	1.25	(0.88,1.76)	0.213	
YP thinks indoor tanning using sun bed/sun lamp can cause skin cancer			0.003	943
no	reference			
yes	0.36	(0.16,0.83)	0.016	
don't know	0.70	(0.27,1.84)	0.472	
**body image**				
YP is trying to change skin colour (0=no/1=yes)	0.90	(0.60,1.35)	0.615	731
YP is trying to change hair colour (0=no/1=yes)	0.89	(0.55,1.43)	0.627	731
YP has been hurt because of their skin colour (0=no/1=yes)	0.74	(0.12,4.55)	0.750	774
YP has been called names because of their skin colour (0=no/1=yes)	1.08	(0.34,3.38)	0.899	780
YP has seen someone bullied because of their skin colour (0=no/1=yes)	0.96	(0.70,1.30)	0.777	774
YP's teachers treat everybody the same, regardless of skin colour (0=agree/1=disagree)	0.76	(0.35,1.62)	0.473	684

[a]Adjusted for age and sex.

Supplementary Table S11. Association of socio-demographic, pigmentation-related and sun exposure factors with indoor tanning using spray tan or self-tanning lotions amongst ALSPAC young people (YP) who like to tan.

exposure	OR[a]	95% CI	p-value	N
**sex** (0=female/1=male)	0.05	(0.03,0.09)	$< 10x^{-26}$	1930
**age** (years)	0.99	(0.82,1.19)	0.908	1930
**mother's social class** (0=manual/1=non-manual)	0.87	(0.63,1.20)	0.401	1598
**father's social class** (0=manual/1=non-manual)	0.73	(0.58,0.91)	0.006	1704
**mother's education**			0.004	1814
< O level	reference			
O level	0.75	(0.56,1.01)	0.060	
> O level	0.62	(0.46,0.82)	0.001	
**skin colour**			$6.23x10^{-7}$	1929
very fair	reference			
fair	0.60	(0.44,0.82)	0.001	
olive	0.38	(0.26,0.54)	$7.94x10^{-8}$	
brown	0.35	(0.17,0.71)	0.004	
**eye colour**			0.348	1926
blue	reference			
green	0.88	(0.67,1.16)	0.363	
grey	0.92	(0.50,1.72)	0.804	
brown	0.79	(0.62,1.03)	0.077	
other	1.18	(0.73,1.92)	0.495	
**natural hair colour at 18 years old**			0.045	1927
red	reference			
blonde	0.68	(0.35,1.33)	0.260	

light brown	0.48	(0.25,0.92)	0.027	
dark brown	0.51	(0.26,0.98)	0.042	
black	0.96	(0.11,8.48)	0.974	
other	0.75	(0.27,2.08)	0.587	
**YP has freckles**			0.006	1926
no	reference			
yes, a few	1.41	(1.12,1.77)	0.003	
yes, many	1.47	(1.06,2.04)	0.019	
**tanning ability**			5.10×10^{-9}	1923
always burns never tans	reference			
burns easily rarely tans	0.16	(0.06,0.46)	0.001	
doesn't change	0.21	(0.07,0.66)	0.008	
tans easily rarely burns	0.09	(0.03,0.26)	5.28×10^{-6}	
always tans never burns	0.12	(0.04,0.37)	2.41×10^{-4}	
**sun burning in the past 2 years**			0.290	1868
never	reference			
once	0.89	(0.66,1.20)	0.443	
twice	0.78	(0.58,1.06)	0.112	
3 times	0.86	(0.58,1.26)	0.432	
4 times	1.17	(0.74,1.85)	0.495	
5 times or more	1.23	(0.73,2.10)	0.436	
**sun protection** (0=no/1=yes)				
never uses protection	0.78	(0.51,1.22)	0.277	1928
wears hat	1.12	(0.87,1.43)	0.377	1928
wears clothing	0.97	(0.76,1.24)	0.791	1928
wears sunblock	1.73	(1.07,2.81)	0.027	1928
avoids the sun	1.03	(0.71,1.50)	0.872	1928
**sunblock factor**			0.074	1798

lower than 15	reference			
15-24	0.63	(0.42,0.95)	0.027	
25-49	0.58	(0.39,0.87)	0.008	
50 or higher	0.62	(0.37,1.07)	0.086	
**skin cancer** (0=no/1=yes)				
YP has family member diagnosed with skin cancer	1.11	(0.82,1.50)	0.492	1925
**beliefs about tanning** (0=no/1=yes)				
YP believes indoor tanning helps prevent sunburn			2.57×10^{-4}	1926
no	reference			
yes	1.84	(1.35,2.51)	1.19×10^{-4}	
don't know	0.92	(0.69,1.22)	0.544	
YP thinks indoor tanning using sun bed/sun lamp can cause skin cancer			0.021	1924
no	reference			
yes	1.18	(0.62,2.22)	0.617	
don't know	0.66	(0.31,1.38)	0.266	
**body image**				
YP is trying to change skin colour (0=no/1=yes)	1.71	(1.20,2.42)	0.003	1537
YP is trying to change hair colour (0=no/1=yes)	2.10	(1.39,3.15)	3.70×10^{-4}	1537
YP has been hurt because of their skin colour (0=no/1=yes)	1.54	(0.33,7.16)	0.508	1604
YP has been called names because of their skin colour (0=no/1=yes)	1.34	(0.58,3.08)	0.495	1608
YP has seen someone bullied because of their skin colour (0=no/1=yes)	1.11	(0.86,1.44)	0.414	1599
YP's teachers treat everybody the same, regardless of skin colour (0=agree/1=disagree)	2.30	(1.21,4.37)	0.011	1478

[a]Adjusted for age and sex.

Supplementary Table S17. Association of indoor tanning using sprays or self-tanning lotions with HIrisPlex-S prediction probabilities of pigmentation traits in ALSPAC young people (YP) (N = 1489).

HIrisPlex-S probabilities	OR[a]	95%	p-value
PBlueEye	1.12	(0.82,1.52)	0.477
PIntermediateEye	1.14	(0.22,5.99)	0.879
PBrownEye	0.87	(0.62,1.22)	0.415
PBlondHair	0.86	(0.51,1.45)	0.580
PBrownHair	0.97	(0.51,1.84)	0.932
PRedHair	2.15	(0.95,4.85)	0.064
PBlackHair	0.36	(0.09,1.49)	0.161
PLightHair	1.41	(0.86,2.31)	0.176
PDarkHair	0.71	(0.43,1.17)	0.176
PVeryPaleSkin	5.16	(1.31,20.32)	0.019
PPaleSkin	1.49	(0.73,3.05)	0.277
PIntermediateSkin	0.57	(0.31,1.07)	0.078
PDarkSkin	0.03	(2.37×10^{-4},4.10)	0.164
PDarktoBlackSkin	0.06	(5.66×10^{-9},6.16×10^{5})	0.731

[a]Adjusted for age, sex and 10 PCs.
